# Effect of microwave (MW)-subcritical extraction on oil recovery, oxidative stability, and lipid types from *Katsuwonus pelamis* livers

**DOI:** 10.1016/j.fochx.2024.101351

**Published:** 2024-04-04

**Authors:** Wenjie Wang, Yuliang Xiao, Yicheng Ding, Yihong Li, Yihua Zhu, Xuxia Zhou

**Affiliations:** a College of Food Science and Technology, Zhejiang University of Technology, Hangzhou 310014, China; b Zhejiang Key Laboratory of Green, Low-carbon and Efficient Development of Marine Fishery Resources, Hangzhou 310014, China; c National R&D Branch Center for Pelagic Aquatic Products Processing (Hangzhou), Hangzhou 310014, China; d College of Biotechnology and Bioengineering, Zhejiang University of Technology, Hangzhou 310014, China

**Keywords:** Tuna liver oil, ω-3 PUFA, MW pretreatment, Subcritical fluid extraction, Dimethyl ether, Lipidomic, Phase separation

## Abstract

*Katsuwonus pelamis* is a tuna species mostly sold for canned fillets, its livers were lack of utilization. This study thus investigated an oil production method combining microwave (MW) pretreatment and subcritical dimethyl ether (SDME) in aim to reach improved efficiency and oil quality. The heating characteristics from different MW powers (400, 600, and 800 W) were evaluated, and SEM showed MW having hydrolysis effect on matrix lipoprotein, the fortified recovery rate was also found. Under the MW-SDME condition with 600 W power, 1:5 solid-to-liquid ratio, and 100 min, the recovery reached 93.21% in maximal (SDME ∼50%). To further improve quality, MW powers was noticed affecting lipid types, fatty acid composition, and oxidative stability of produced oils. 1286 lipid types (mostly glyceride and phospholipid-type) were identified, while higher MW lowered the emulsifying phospholipids prompting phase separation. Several oxidation indexes consistently increased with the rising MW power, GC–MS suggested 400 W for higher DHA.

## Highlights

• The MW-SDME method promote oil release with 93.21% recovery.
• Stronger MW power lower the proportion of emulsifying phospholipids.
• 400 W-SDME is suitable for DHA-oils production.
• 600 W-SDME showed the best performance for recovery and overall quality.

## Graphical abstract

**Figure:**
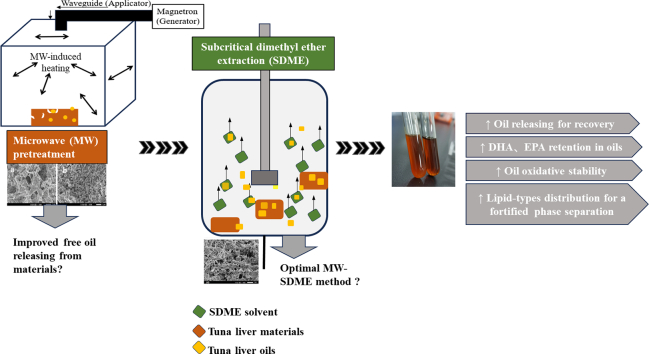

## 1 Introduction

Tuna (*Katsuwonus pelamis)* was globally popular in terms of high ω-3 oils and fish proteins (Song et al., 2020). The annual catch of tuna in the world has exceeded 4 million tons, accounting for 3% of the total output (FAO, 2022). Recently, growing attention has been given to the use of fish by-products (Nikoo, Regenstein, & Yasemi, 2023; Stevens, Newton, Tlusty, & Little, 2018). 70% of inedible parts of tuna could be collected from a commercial production line, including head, skin, and bone (Sasidharan & Venugopal, 2020). Tuna livers are considered a critical resource for dietary ω-3 polyunsaturated fatty acids (PUFA) like EPA, (C_20:5_) and DHA (C_22:6_) (Fakhri et al., 2022), possessing high antioxidant and anti-inflammatory activity. They were frequently reported having health benefits including blood viscosity, blood pressure, and blood sugar reduction, prolonging risk of cardiovascular disease (CVD) (Chen, Jayachandran, Bai, & Xu, 2022; Kapoor, Kapoor, Gautam, Singh, & Bhardwaj, 2021; Oppedisano et al., 2020).

At present, the common fish oil production methods involved cooking extrusion, solvent extraction with alkali hydrolysis, enzymatic hydrolysis and novel fluid systems such as supercritical CO_2_ (Rubio-Rodríguez et al., 2012). Among, traditional cooking extrusion has only 1–12% oil yield (García-Moreno et al., 2014). Solvent extraction and alkaline extraction under atmosphere had a higher oil yield, but concerns on environmental pollution from chemical residue suppressed the application (Hoyos Concha & Bonilla, 2018). The key limits for oil extraction from materials is the strong affinity between matrix proteins and oil droplets. Even though biological and chemical pretreatments could break the chemical bondage between matrix proteins and oils to a certain extent. But the high cost, weak thermal stability of enzymes, and solvent concerns make them hard to use in large-scale production. Heating is another effective way for oils releasing fortification, while the long-time cooking or drying in conventional ways consumed huge energy and made produced oils more liable to oxidative rancidity (Pudtikajorn & Benjakul, 2020). Microwaves (MW) might be a solution, as it could form an instant volumetric heating by initiating vibrations of waters molecules under MW induced electromagnetic field. It was an eco-friendly pretreatment for collecting various edible oils (Mwaurah et al., 2020). Some studies showed that MW-assisted solvent extraction improved the extraction efficiency with reduced usage of organic solvents (Bruno, Kudre, & Bhaskar, 2019; Costa & Bragagnolo, 2016; Ramalhosa et al., 2012; Sathivel, Prinyawiwatkul, King, Grimm, & Lloyd, 2003).

Moreover, novel solvent systems such as supercritical CO_2_ extraction attracted attentions for extracting polysaccharides, oils, and polyphenols from plant materials mostly, its application on perishable aquatic food materials is limited, considering high operation cost and serious protein denaturation from extremely high heat (over 100 °C) and pressure (Rubio-Rodríguez et al., 2008). In this case, subcritical fluid with much lower pressure and heat became a preferred alternative (Guo, Wan, Huang, & Wei, 2021). Subcritical solvents remain as gas phase under the atmosphere, while liquefy at slight pressure and mild temperature. This phenomenon made subcritical extraction able to auto-separation after releasing pressure, and the complete solvent removal effect was also realized. Subcritical dimethyl ether (SDME) with a high polarity exhibited great extraction efficiency directly on moist aquatic food materials, especially the neutral lipids (Catchpole et al., 2009; Catchpole et al., 2010). DME remains in a subcritical state under 126.95 °C/5.33 MPa with a wide practical range. Considering its extremely low melting point (−24.8 °C), no SDME would be left in the final product theoretically (Ma et al., 2019). DME itself was also proven with low biological toxicity and has was allowed to be used as a food processing aid in Europe, Australia, and New Zealand (Bauer & Kruse, 2019; Kanda, Katsube, & Wahyudiono, & Goto, 2021). Besides, our former works summarized an high-yield SDME condition for tuna livers with optimal stirring speed, temperature, and pressure, already, confirmed SDME having similar quality as supercritical CO_2_ (Fang et al., 2018) and better efficiency over other conventional methods (Fang et al., 2019). This paper therefore expected a finding on synergetic effect from MW and SDME in terms of oil recovery rate and improved oil quality.

The paper optimizes a MW-SDME extraction condition with a high oil recovery rate by a systematic design. Then the Lipid-types profile, fatty acid composition, oxidative stability of produced oils was evaluated by main factors on quality fortification, morphology changes of liver materials would provide information on supporting oil releasing fortification from the application of MW-SDME techniques.

## 2 Materials and methods

### 2.1 Materials and reagents

The freshly collected tuna (*Katsuwonus pelamis*) livers were supplied by Ning Bo Today Food Co. (Zhejiang, China) from the same batch of canning processing. Endogenous enzymes were all inactivation prior to rapid freezing at −40 °C. Frozen samples were then transported at −18 °C vehicle, arrived the laboratory within same day. All samples were immediately transferred and stored in a − 30 °C freezer room. Samples were set in a 4 °C refrigerator overnight for thawing before all processing and analysis.

Boron trifluoride (BF_3_) in methanol (13–15%) was provided by Adamas-Beta Co. (Shanghai, China). Standards of 37 individual fatty acid methyl esters were purchased from ANPEL Laboratory Technologies Co. (Shanghai, China). HPLC-grade hexane was acquired from Aladdin Reagent Co. (Shanghai, China). Analytical-grade chloroform, methanol, n-hexane, ether, isopropanol, isooctane, acetic acid, sodium hydroxide, sodium chloride, potassium iodide, sodium thiosulfate, and *p*-anisidine were provided from Sinopharm Chemical Reagent Co. Technical-grade DME gas (99.95% purity) was purchased from Jingong Special Gas Co. (Hangzhou, China).

### 2.2 MW-assisted subcritical fluid extraction process

MW-assisted subcritical fluid extraction process was a two-step operation. 200 g of liver samples were placed in a glass dish inside of the microwave machine under MW power at 400, 600, and 800 W for a constant time based on the heating characteristics results. Then, the microwave-pretreated sample was immediately subjected to a subcritical extraction, conducted by a 3-L type CBL Subcritical fluid extractor manufactured by Henan Subcritical Biological Technology Co. (Henan, China). Subcritical dimethyl ether (SDME) extraction was carried out with a similar setting from previous work (Fang et al., 2018). 500 g of MW-pretreated livers were placed in a nylon bag inside the subcritical extraction tank. Based on the targeted solid-liquid ratio (*w*/*v*), a certain volume of SDME was merged into the extraction tank from the liquid tank under pressure at 0.5–0.8 MPa which was chosen based on a previous study (Fang et al., 2020). The stirring speed at 925 rpm and heating at 30 °C were applied based on former study (Fang et al., 2018), the SDME processing last for a maximum of 120 min based on our preliminary works. After each operation, the released pressure would recover the DME gas under the support of a vacuum pump. The produced fish oil could be independently collected from the recovery tank below the extraction tank. To ensure sufficient oil collection, the tank walls were rinsed with n-hexane for two times, after centrifugation and condensation, the combined final fish oil would be dehydrated with anhydrous Na_2_SO_4_ powders. The produced oils were weighed for calculating oil recovery and stored at −30 °C freezer room prior to following tests.

### 2.3 Experimental design

To better evaluate the effect of the MW-SDME method on oil recovery, the microwave power (X_1_), extraction time (X_2_), and solid-to-liquid ratio (X_3_) were studied as independent variables under the response surface experiments targeting high extraction efficiency. As the appropriate solid-liquid ratio can promote the oil extraction efficiency (Bu et al., 2021), extraction time and solid-liquid ratio are among the top factors based on preliminary work, 600 W, 90 min and 1:5 was selected as the central points of the variable factor for X_1_, X_2,_ and X_3_, respectively in the RSE design. Table 1 illustrates the variables and levels for all three factors.

**Table 1:** Factors and levels under the MW–SDME method.

**Independent variables**	**Symbol**	**Variable levels**		
		**-1**	**0**	**1**
Microwave power (W)	X_1_	400	600	800
Extraction time (min)	X_2_	60	90	120
solid-to-liquid ratio (g/mL)	X_3_	1:3	1:5	1:7

### 2.4 Oil recovery evaluation

Oil recovery was evaluated by a modified method from (Ramalhosa et al., 2012). It is based on the weight percentage of extracted oils to the total oils of the materials. The total oils of tuna livers were collected by Folch extraction and vacuumed condensation.

$$\mathrm{Oil recovery rate}\;\left(\%\right)=\frac{\text{Weight of extracted oils}\;\left(g\right)}{\text{Weight of Total oils of tuna livers}\;\left(g\right)}\times 100\%.$$

### 2.5 Chemical analysis of oils

#### 2.5.1 Oxidative stability

Several oxidative stability indicators of produced oils were measured including peroxide value (POV), acid value (AV), total oxidation value (TOTOX), p-anisidine value (*p*-AV), and iodine number (IV).

POV value was determined by AOCS official method Cd 8–53(AOCS, 2003), indicating primary oxidation with the increased formation of hydroperoxides. The results were expressed as units of meq (O_2_) /kg.

AV value of fish oil was determined by the titration method described by Rasel Molla et.al (Rasel Molla, 2016), illustrating the free fatty acids and rancidity levels of oils at the late stage of oxidation with the decomposition of triglycerides. The results were expressed as units of mg (KOH) /g.

The *p*-AV value was determined by UV–visible spectrophotometry with reference to Hwang et al. (2017), assessing the secondary oxidation level of oils with the presence of aldehydes and ketones.

TOTOX was a measure of oils' overall oxidation profile and calculated from the POV and *p*-AV with the formula as below:

TOTOX=2POV+p−AV.

Besides, IV reflects the oxidation degree of unsaturated fats in produced oil (Novizar Nazir & Sayuti, 2017). It was determined by AOCS official method Cd 1d-92(AOCS, 2022), the results were expressed as the unit of g/100 g.

#### 2.5.2 Fatty acids distribution

It was determined by the methyl esterification method with modification (Chakraborty & Joseph, 2015). 0.1 g of oil samples was added with 2 mL 2% NaOH-methanol solution, set in a 65 °C water bath for 3 min. After cooling, 2 mL 14% BF_3_-methanol solution was added and agitated for 3 min at a 65 °C. After cooling, 2 mL n-hexane and 2 mL saturated NaCl solution were added, agitated, and set for phase separation at ambient temperature, the supernatant was collected and went through anhydrous Na_2_SO_4_ for dehydration. The dehydrated oils were filtered (0.23 μm) before the GC–MS analysis.

The fatty acid methyl esters (FAME) in oils were analyzed by a Thermo Fisher Scientific type TRACE 1300 gas chromatographer (MA, U.S.A.) equipped with an Agilent Technologies type HP-INNOWax MS capillary column (60 m × 0.32 mm × 0.25 mm) (CA, U.S.A.). Helium was used as the carrier gas with constant current mode, in which the flow rate 2 mL/min. The column temperature follows the following gradient setting, as the initial temperature of 90 °C was maintained for 5 min, raised to 200 °C at a rate of 15 °C/min, then raised to 240 °C at a rate of 1 °C/min and kept at 240 °C for 10 min. The injection volume was 1 μL with no split mode. The MS was in EI mode (70 eV) with a 0.2 scan per second, interval over a 35–450 *m*/*z* range. Most of the fatty acid methyl esters could be identified and quantitated by comparison of retention time and relative percentages of detected peak areas to the known standards' peak areas of 37 FAME mix.

#### 2.5.3 Lipid-types profile

To verify the lipid-types profiles in terms of composition and glycerophospholipid molecular species, which were separated by Nexera LC-30A ultra-high performance liquid chromatography (UHPLC) system and detected by electrospray ionization (ESI) positive and negative ion modes. LC-MS/MS method was used to determine instrument parameters with 3 μL of injection volume. The setting was operated as column temperature 45 °C, 300 μL/min flow rate. Mobile phase A uses 10 mM ammonium formate in 60% (*v*/v) acetonitrile aqueous solution, and phase B applies 10 mM ammonium formate in 10% (v/v) acetonitrile isopropanol solution. The gradient elution follows: 0–2 min, B maintained at 30%; 2–25 min, B changed linearly from 30% to 100%; 25–35 min, B maintained at 30%. The samples were placed in an automatic sampler at 10 °C throughout the analysis. The samples were separated by UHPLC and analyzed by Thermo Scientific Q Exactive plus type mass spectrometer (MA, U.S.A.). The ESI source conditions: Positive: Heater Temp 300 °C, Sheath Gas flow rate 45 arb, Aux Gas Flow rate 15 arb, Sweep Gas Flow rate 1arb, spray voltage 3.0KV, Capillary Temp 350 °C, S-Lens RF Level 50%. MS1 scan ranges 200–1800. Negative: Heater Temp 300 °C, Sheath Gas Flow rate 45 arb, Aux Gas Flow Rate 15 arb, Sweep Gas Flow Rate 1arb, spray voltage 2.5KV, Capillary Temp 350 °C, S-Lens RF Level 60%. MS1 scan ranges 250–1800. This project uses a non-targeted lipidomics analysis platform based on the UPLC-Orbitrap MS system, combined with Thermo Scientific LipidSearch software (MA, U.S.A.) for lipid identification and data preprocessing.

### 2.6 Microstructural observation on morphological changes on tuna livers

Referring to Ye et al. (2021), different freeze–dried tuna liver samples (untreated, MW, MW-SDME) were fixed by the conductive carbon paste and placed under the scanning electron microscope (SEM) sample stage, the gold plating was performed with a vacuum ion sputtering instrument, and then scanning was performed.

### 2.7 Statistical analysis

Statistical analysis in this study was performed by SPSS statistics software version 20.0 (IL, U.S.A.). All experiments were conducted in triplicates and data was expressed as mean ± SD (*n* = 3) and significant differences among treatments were determined by one-way ANOVA with *Tukey*'s test (*P* < 0.05).

## 3 Results

### 3.1 Heating characteristics of samples under different MW powers

**Fig. 1:**
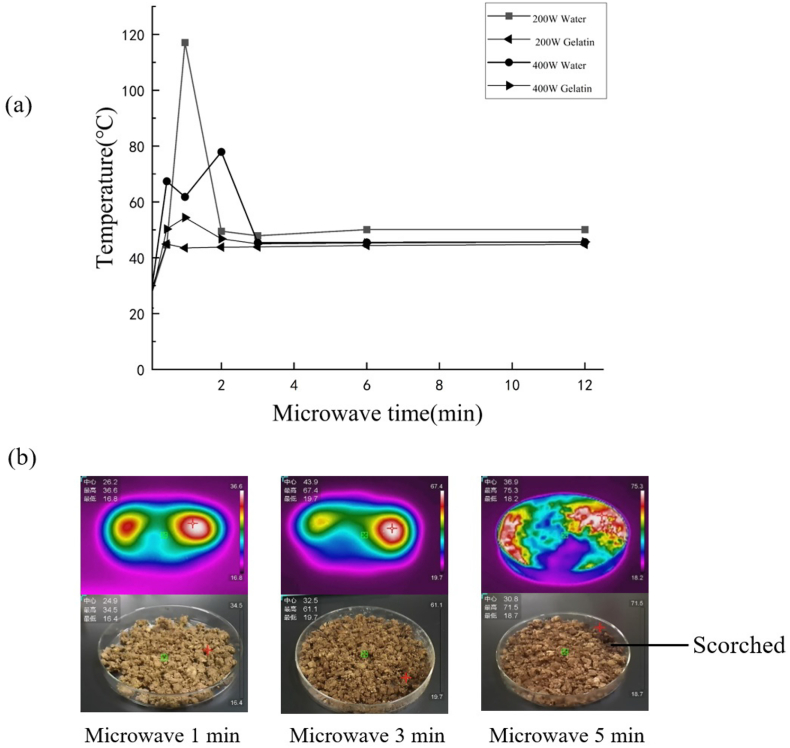
(a) Heating characteristics across times for model materials (Water and gelatin) under 200 and 400 W MW powers, (b) Heatmap for MW-treated tuna liver materials with 400 W MW power at 1-, 3- and 5-min heating time-point.

Based on our results in Fig. 1a, temperatures of both model materials fluctuated significantly under both MW powers in the first 3 min, then reached a consistent heating. However, the degree of temperature fluctuations (up to 20–80 °C) was found to be more severe in water rather than gelatin paste (23%), confirming that high content of polar composition (i.e., Water) was able to induce more unstable heating on materials in the first 3-min heating. Besides, the gelatin model materials reached their temperature consistency just after 1-min heating, illustrating a faster heat balance. In addition, the increase of applied MW powers facilitated reaching heat balance without influencing the final heating temperature. To clarify the heating characteristics of tuna liver samples, 400 W was thus applied for heating pretreatment for 1-, 3- and 5-min (Fig. 1b). It was found that the longer MW treated times go, the more uniform heating it would present. Since MW heating over 5 min exhibited an obvious edge burn, 3-min MW pretreatment was chosen for the subsequent experiments.

### 3.2 Optimization of the MW-SDME oil extraction

Based on our preliminary studies, the matrix lipoprotein was broken under MW pretreatment and the releasing effect of oils from the materials was increased. It was important to evaluate the key factors influencing the extraction efficiency of the MW-SDME method. Based on our results from 3^2^ response surface experiments shown in Table 2, the oil recovery rate from tuna livers under different MW-SDME extraction conditions was in the range of 56.50–95.31%. The experimental results were also fit by quadratic multiple regression, the equation was as follows:

**Table 2:** Oil recovery rate under different MW-SDME methods.

**Trials**	**MW–SDME conditions**				**Oil recovery rate (%)**	
	**X**_**1**_	**X**_**2**_		**X**_**3**_		
1	400	60		1:5	77.54	
2	800	60		1:5	67.50	
3	400	120		1:5	70.66	
4	800	120		1:5	82.37	
5	400	90		1:7	68.24	
6	800	90		1:7	56.50	
7	400	90		1:3	82.40	
8	800	90		1:3	83.45	
9	600	60		1:7	60.28	
10	600	120		1:7	58.08	
11	600	60		1:3	75.95	
12	600	120		1:3	85.15	
13	600	90		1:5	95.31	
14	600	90		1:5	90.96	
15	600	90		1:5	89.64	
16	600	90		1:5	92.58	
17	600	90		1:5	90.43	

**Source**	**Sum of squares**	**Df**	**Mean square**	**F-value**	***P*-value**	**Significance**
Model	0.25	9	0.028	48.72	<0.0001	**
X _1_	1.02 × 10^−3^	1	1.02 × 10^−3^	1.79	0.2226	
X _2_	2.81 × 10^−3^	1	2.81 × 10^−3^	4.96	0.0613	
X _3_	0.088	1	0.088	154.98	<0.0001	**
X _1_ × _2_	0.012	1	0.012	20.86	0.0026	**
X _1_ × _3_	4.04 × 10^−3^	1	4.09 × 10^−3^	7.22	0.0312	*
X _2_ × _3_	3.25 × 10^−3^	1	3.25 × 0^−3^	5.73	0.0479	*
X _1_^2^	0.022	1	0.022	38.96	0.0004	**
X _2_^2^	0.042	1	0.042	74.61	<0.0001	**
X _3_^2^	0.060	1	0.06	105.06	<0.0001	**
Residual	3.97 × 10^−3^	7	5.67 × 10^−4^			
Lack of fit	1.95 × 10^−3^	3	6.51 × 10^−4^	1.29	0.3916	
Pure terror	2.02 × 10^−3^	4	5.04 × 10^−4^			
*R*^*2*^	0.9843					
CV%	3.05					

Noted MW power (X_1_, W), extraction times (X_2_, min), and solid-liquid ratio (X_3_, g/mL).

$$Y=0.92–0.011\times X_{1}+0.019\times X_{2}+0.1\times X_{3}+0.054\times X_{1}\times X_{2}+0.032\times X_{1}\times X_{3}+0.028\times X_{2}\times X_{3-}0.072\times {X_{1}}^{2}–0.1\times {X_{2}}^{2}-0.12\times {X_{3}}^{2}.$$

From the statistical significance results in Table 2, it was shown that the whole model is effective with *P* < 0.0001, indicating that the fitted equation tests very well and the error is negligible. Among all three factors, the oil recovery rate from tuna livers was mostly influenced in ranks as follows: solid–liquid ratio (X_3_) > extraction time (X_2_) > microwave power (X_1_). In the subcritical system of static mode, the ratio of sample to solvent may be a key factor (Halim, Abidin, Siajam, Hean, & Harun, 2021). The solute-solvent equilibrium concentration and the solubility of the compound have a great influence on the extraction ability (Teo, Tan, Yong, Hew, & Ong, 2010). Adequate extraction time is conducive to the dissolution of oil, while the microwave is only a way of pretreatment of raw materials. Extraction mainly occurs in the subcritical system. Compared with the parameters of the subcritical system, the contribution of the pretreatment method to the recovery of fish oil may not be so large. In the subcritical system, the solid-liquid ratio had the greatest influence on oil extraction, followed by extraction time, which was consistent with the study of (Cai et al., 2022). *R*^2^ and *R*^2^_adj_ were 0.9843 and 0.9641, respectively, indicating a high correlation between the predicted value and the experimental value. The coefficient of variation (C.V.) was at only 3.05%, indicating that the equation had high confidence and was able to represent the changes in response values.

The optimum extraction conditions predicted by the model were as follows: microwave power 619.41 W, extraction time 95.63 min, solid-liquid ratio 0.28 g / mL. Under the optimal conditions, the maximum extraction efficiency was 94.4%. Considering the convenience of the actual experiment, the optimal experimental variables were determined as follows: microwave power 600 W, extraction time 100 min, solid-liquid ratio 1: 4 g/mL. Under these conditions, the average recovery rate of fish oil obtained by three experiments was 93.21 ± 0.92%, which was close to the predicted value (94.4%), and the relative error was 1.28%. This result shows the accuracy and effectiveness of the response model.

### 3.3 Evaluation of extraction efficiency and oxidative stability under different MW powers

**Fig. 2:**
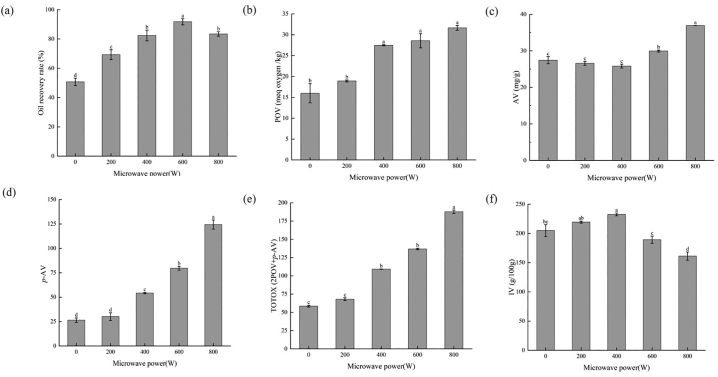
Effect of MW powers (0, 200 W, 400 W, 600 W and 800 W) on (a) oil recovery rate, (b) peroxide value (POV), (c) acid value (AV), (d) *p*-anisidine value (*p*-AV), (e) Total oxidation value (TOTOX), and (f) iodine number (IV) for tuna liver oils produced from different MW powers under MW-SDME methods. Noted TOTOX was calculated from the formula: 2POV + *p*-AV; Different lower letters above histograms in each figure illustrated significant differences among treatments (*P* < 0.05).

Based on the optimization study, a 90-min extraction time and of 1:4 solid-liquid ratio were selected for the following systematic comparison of extraction efficiency and oxidative stability under different MW powers (untreated, 200, 400, 600, and 800 W) within MW-SDME treatment, The oil recovery rate and oil oxidation properties including peroxide value (POV), acid value (AV), *p*-anisidine value (*p*-AV), total oxidation value (TOTOX) and iodine value (IV) of produced fish oil were tested (Fig. 2).

As shown in Fig. 2a, the SDME method has reached 50% oil recovery rate. However, in the study of oil extraction from tuna by-products(Novizar Nazir & Sayuti, 2017), the extraction rate of the wet refining method was only 12.8%, the solvent method was 8.49%, and the acid silage method was 6.16%. Cutajar et al. (2022)showed that the maximum yield of Atlantic bluefin tuna oil extracted by enzymatic method was 29%. Compared with the conventional oil extraction method, the subcritical extraction method seems to have certain advantages, which are similar to the results of Fang et al. (2019). MW pretreatment elevated the oil release, and the maximum oil recovery rate reached 93.21% at 600 W. The oil recovery rate was improved by increasing microwave power from 200 to 600 W and declined by increasing microwave power to 800 W. The high microwave power makes the temperature rise quickly, which leads to the partial thermal decomposition of oil to a certain extent (Chen, Du, Zu, Yang, & Wang, 2016; Rezvankhah, Emam-Djomeh, Safari, Askari, & Salami, 2019).

POV is the primary product of lipid oxidation during the processing and storage of aquatic products (Šimat et al., 2020). As shown in Fig. 2b, the POV value of fish oil from SDME without MW pretreatment was 15.99 meq/kg. With the increase in microwave power, the POV value of fish oil overall showed an upward trend. At low MW power (200 W), there was no significant difference with the MW untreated group. At the same time, significance was shown between 200 W and medium-to-high MW power groups (400 W, 600 W, 800 W). The increase in POV value may be due to the thermal degradation of fatty acids and the accumulation of peroxides caused by the attack of free radicals on unsaturated fatty acids (Ji, Liu, Shi, Wang, & Wang, 2019).

AV is another important index interpreting the level of lipid oxidation regarding secondary products as free fatty acids (FFA). As shown in Fig. 2c, the AV value of fish oil extracted from the SDME group was 27.45 mg/g. Overall, AV value increased with the rise of MW powers. But there is no significant difference between it with low-to-medium MW powers including 200 and 400 W. Higher MW power (600 W) significantly increases the AV to 29.95 mg/g and above. The increase in AV is due to the cleavage of the existing bonds between glycerol and fatty acids and the release of free fatty acids in the oil (Suri, Singh, & Kaur, 2022).

The *p-*AV value emphasizes the content of produced aldehydes (α, β-unsaturated aldehydes) from lipids oxidation, as the main flavor-related secondary oxidation products, it was also commonly used for interpreting the degree of lipid oxidation (Faas, Röcker, Smrke, Yeretzian, & Yildirim, 2020). Based on Fig. 2d, the *p-*AV value of fish oil from SDME without MW pretreatment was 26.48 and reached 30.19 for the 200 W-SDME method, there is no significant difference between the two groups. While the *p-*AV value increased significantly (*P* < 0.05) from 400 W to 800 W. The *p*-AV value increases with the increase of microwave power, which is similar to the results of Suri et al.(Suri et al., 2022). The increase in *p*-AV value is related to the degradation of primary hydroperoxides into non-volatile carbonyl compounds(Hashemi et al., 2017).

According to Fig. 2e, the consistent trend as *p-*AV was found in TOTOX value which related to the presence of hydroperoxides, aldehydes, ketones, and other oxidized compounds in the oils, mainly produced by PUFA degradation under high temperature, oxygen, metal compounds and light (Rubio-Rodríguez et al., 2012). Compared with the other groups, the 800 W group reached the highest oxidation state, while there was no significant difference between the 600 W group and the 400 W group.

Besides, Fig. 2f illustrated that IV of oils from different MW powers under the MW-SDME method was in the range of 168–234 g/100 g, these values were slightly higher than reported carp viscera oils (83–94 g/100 g)(Flavia Pop, & Anca dumuta, Zorica Vosgan., 2013) and tuna fish oils (175–185 g /100 g) (Sahena et al., 2013)due to difference of fish species, harvest seasons and material parts. Besides, as MW induced heating on materials, IV of oils from heat-pretreated fish materials could increase due to the progress of oxidation. A similar trend was also found by(Tanko Bako & Awulu, 2017). Among our treatments, it was found that 400 W-SDME reached a significantly higher IV over the SDME group and 600 W- and 800 W-SDME groups. It indicated that 400 W as MW pretreatment in MW-SDME methods contributed to a higher degree of unsaturation (supported by UFAs results in session 3.4) in produced oils, suggesting its improved oil quality.

In summary, 400 W was a suggested MW pretreatment condition in consideration of higher oil recovery and better oil stability.

### 3.4 Evaluation of fatty acids distribution under different MW powers

As presented in Table 3, the fatty acids distribution in produced oils from different MW powers under MW-SDME was provided. A total of 18 individual fatty acids were detected in produced tuna liver oils. Compared with the fatty acid composition of skipjack tuna eye socket oil(Pudtikajorn & Benjakul, 2020), the content of polyunsaturated fatty acids in our results is fairly low, but monounsaturated fatty acids are higher, this might be caused by the variation among tuna types and different tissues. From our findings, saturated fatty acids (SFA) accounted for 37.54–44.37%, monounsaturated fatty acids (MUFA) accounted for 24.03–33.36%, polyunsaturated fatty acids (PUFAs) accounted for 27.98–38.43%. In specific, detected SFAs were mainly palmitic acid and stearic acid, accounting for 22–27% and 10–11% of the total fatty acids, respectively. MUFAs in produced oils are mainly oleic acid (15–21%). Among PUFA, DHA and EPA occupied 30–42% of the total fatty acids, followed by arachidonic acid (3.49–4.54), etc.

**Table 3:** Effect of different microwave powers (0, 200 W, 400 W, 600 W, and 800 W) on the fatty acid composition of oil from tuna viscera extracted by microwave–assisted subcritical dimethyl ether extraction (MW-SDME).

**Fatty acids**	**Relative content for individual fatty acids (%)**				
	**untreated (0 W)**	**200 W**	**400 W**	**600** **W**	**800** **W**
Myristic acid (C14:0)	1.76 ± 0.21^a^	1.86 ± 0.03^a^	1.73 ± 0.01^a^	1.76 ± 0.02^a^	1.76 ± 0.09^a^
Pentadecanoic acid (C15:0)	1.00 ± 0.13^a^	1.00 ± 0.04^a^	1.07 ± 0.02^a^	1.08 ± 0.01^a^	0.97 ± 0.06^a^
Palmitic acid (C16:0)	27.46 ± 1.69^a^	25.86 ± 0.74^a^	22.04 ± 0.82^b^	27.54 ± 0.65^a^	25.7 ± 0.68^a^
Palmitoleic acid (C16:1)	3.82 ± 0.31^c^	4.32 ± 0.10^ab^	3.97 ± 0.02^bc^	4.37 ± 0.07^ab^	4.68 ± 0.19^a^
Heptadecanoic acid (C17:0)	1.87 ± 0.15^a^	2.03 ± 0.08^a^	2.06 ± 0.04^a^	2.13 ± 0.14^a^	1.87 ± 0.17^a^
Margaric acid (C17:1)	0.74 ± 0.06^c^	0.82 ± 0.03^bc^	0.80 ± 0.01^bc^	0.88 ± 0.01^b^	0.98 ± 0.06^a^
Stearic acid (C18:0)	10.05 ± 0.77^b^	11.07 ± 0.15^ab^	10.31 ± 0.19^b^	11.48 ± 0.69^a^	10.07 ± 0.59^b^
Elaidic acid (C18:1, trans)	17.87 ± 0.44^b^	17.89 ± 0.31^b^	15.36 ± 0.32^c^	18.13 ± 0.03^b^	21.83 ± 0.8^a^
Oleic acid (C18:1, cis)	2.35 ± 0.30^b^	2.84 ± 0.08^a^	2.79 ± 0.05^a^	2.96 ± 0.14^a^	2.82 ± 0.22^a^
Linoleic acid (C18:2, ω-6)	1.34 ± 0.09^a^	1.19 ± 0.03^b^	1.24 ± 0.01^a^	1.25 ± 0.02^a^	1.16 ± 0.08^b^
γ-linolenic acid (C18:3, ω-6)	0.36 ± 0.04^c^	0.66 ± 0.02^a^	0.72 ± 0.01^a^	0.26 ± 0.03^d^	0.55 ± 0.07^b^
α-linolenic acid (C18:3, ω-3)	0.32 ± 0.02^a^	0.28 ± 0.01^b^	0.28 ± 0.01^b^	0.29 ± 0.02^b^	0.26 ± 0.02^b^
Arachidic acid (C20:0)	0.27 ± 0.04^b^	0.36 ± 0.02^a^	0.34 ± 0.01^ab^	0.32 ± 0.05^ab^	0.30 ± 0.04^ab^
Eicosenoic acid (C20:1)	0.83 ± 0.08^b^	1.11 ± 0.03^a^	1.10 ± 0.02^a^	1.05 ± 0.09^a^	1.04 ± 0.11^a^
Eicosadienoic acid (C20:2)	0.33 ± 0.03^ab^	0.34 ± 0.02^ab^	0.38 ± 0.00^a^	0.35 ± 0.03^ab^	0.31 ± 0.04^b^
Arachidonic acid (C20:4, ω-6)	4.12 ± 0.27^b^	4.02 ± 0.06^bc^	4.54 ± 0.06^a^	3.70 ± 0.11^cd^	3.49 ± 0.28^d^
EPA (C20:5, ω-3)	7.26 ± 0.71^a^	6.68 ± 0.24^ab^	6.95 ± 0.24^a^	5.98 ± 0.22^b^	5.95 ± 0.26^b^
DHA (C22:6, ω-3)	18.76 ± 0.96^b^	18.41 ± 1.30^bc^	24.33 ± 1.59^a^	16.40 ± 0.31^bc^	16.25 ± 0.77^c^
**SFA**	42.41 ± 0.35^b^	41.78 ± 0.71^b^	37.54 ± 1.02^c^	44.37 ± 0.18^a^	40.67 ± 0.34^b^
**MUFA**	25.61 ± 0.96^c^	26.92 ± 0.41^b^	24.03 ± 0.35^d^	27.40 ± 0.19^b^	31.36 ± 0.23^a^
**PUFA**	31.98 ± 0.66^b^	31.29 ± 1.06^b^	38.43 ± 1.34^a^	28.23 ± 0.37^c^	27.98 ± 0.25^c^

Noted different letter lower in superscript for results in each line illustrated significant differences among treatments (*P* < 0.05).

There were obvious differences in the fatty acid distribution of produced oils between the MW-treated group and the untreated group. Specifically, MW pretreatment elevated the content of DHA and long-chain MUFAs such as octadecenoic acids, eicosenoic acids, heptadecenoic acids, and palmitoleic acids. Besides, different MW powers affect the content of individual fatty acids. For instance, the fish oils produced by 600 W-SDME have the highest SFA content. With the increase of MW powers, 800 W reached the highest MUFA proportion in the total FAs. It was also noticed that oils produced from 400 W pretreatment remained the most PUFA content in comparison with all other groups. High microwave power may lead to the degradation of unsaturated fatty acids and change the proportion of fatty acids (Suri, Singh, Kaur, Yadav, & Singh, 2020). It might be due to the synergetic effects from both MW pretreatment and fast extraction of SDME, a mild processing was presented due to the proper heating and rapid extraction process. Therefore, 400 W-SDME was suggested in terms of high retention of unsaturated fatty acids in the crude oils.

### 3.5 Evaluation of lipid-types profile under different MW powers

As shown in Table 4, In terms of lipid types, there are a total of 1286 sub-types of lipids detected in produced tuna oil under six main types of lipid components, including glycerophospholipids, glycerolipids, fatty acyls, sphingolipids, saccharolipids and sterol lipids based on the lipidomic analysis. Besides, the lipid profiles for produced tuna liver oils were dominated by polar glycerophospholipids, mostly followed by neutral glycerolipids (TAG, DAG, and MAG) and fatty acyls. They accounted for over 96% of the total lipid components. Based on our results, glycerophospholipids are the most condensed lipid component types in crude tuna liver oils. And results showed that phosphatidylcholine (PC, 38.2–47.4%), triacylglycerol (TAG, 17.1–25.1%), lysophosphatidylcholine (LPC, 9.8–11.5%), diacylglycerol (DAG, 6.9–8.1%) and free fatty acids (FFA, 6.9–9.6%) are the four main lipid types in oils. This finding is consistent with the lipid composition detected in the muscle tissues of bigeye tuna from the previous study (Wang, Xie, & Chen, 2021). In general, MW powers have distinguished effects on PC, TAG, LPC, and FFA. To further estimate the main lipid types in consideration of polarity targeted for future crude oil refinement, our results of HPLC-MS / MS in Table 5 showed that the tuna oil extracted by MW-SDME contained most polar phospholipid (PL) in range of 56.7–66.2%, FFA in range of 6.9–8.1%, MAG in range of 0.32–0.41%, DAG in range of 6.9–9.6%. Regards to neutral lipids which are most desired in this study for producing oils with better separation properties in the following refinement, it was found our oils contained 0.04–0.05% cholesterol (CHO) and 17.1–25.1% TAG. Based on the statistical analysis, significant differences were detected among different MW powers under MW-SDME treatment groups (*p* < 0.05). Most neutral lipids and least PL in oils were realized from the highest MW powers (800 W), and although a slight difference was detected between the MW untreated group and treated groups for FFA content, there is no significant difference found in MAG and CHO content.

**Table 4:** Lipid-type distribution in produced tuna liver oil from different MW–SDME treatments.

**Lipid type**	**Sub–types**	**Amt.**	**Proportion (%)**				
			**0 W**	**200 W**	**400 W**	**600** **W**	**800** **W**
***Glycerophospholipids:***	Phosphatidylcholine (PC)	307	47.4719	46.8034	47.6818	38.2271	45.4727
	Phosphatidylethanolamine (PE)	27	0.6118	0.6925	0.7083	0.3173	0.2237
	Phosphatidylethanol (PEt)	20	1.5595	1.3726	1.6428	1.7152	1.4425
	Phosphatidylglycerol (PG)	80	1.4352	1.4998	1.3220	1.3357	1.1932
	Phosphatidylinositol (PI)	2	0.0020	0.0041	0.0104	0.0045	0.0021
	Phosphatidylmethanol (PMe)	6	0.0812	0.1711	0.0873	0.2949	0.1758
	Phosphatidylserine (PS)	19	0.2544	0.3282	0.2202	0.8337	0.4942
	Phosphatidic acid (PA)	6	0.0453	0.0555	0.0401	0.0670	0.0381
	Platelet activating factor (PAF)	2	0.0327	0.0202	0.0235	0.0925	0.1088
	Cardio phospholipids (CL)	2	0.0103	0.0090	0.0109	0.0007	0.0054
	Cyclophosphatidic acid (cPA)	1	0.0022	0.0045	0.0006	0.0059	0.0018
	Dimethyl phosphatidyl ethanolamine (dMePE)	22	0.4161	0.4111	0.4161	0.3734	0.4278
	Dimethyl lysophosphatidyl ethanolamine (LdMePE)	12	0.7894	0.8944	0.8085	0.9011	1.0645
	Lysophosphatidic acid (LPA)	1	0.0410	0.1264	0.0730	0.2740	0.1260
	Lysophosphatidyl choline (LPC)	84	9.7543	10.5007	10.2416	8.3522	11.4678
	Lysophosphatidyl ethanolamine (LPE)	7	0.2630	0.3295	0.2873	0.1436	0.1317
	Lysophosphatidyl glycerol (LPG)	4	0.1447	0.1736	0.1223	0.2409	0.2421
	**Total**	**602**	**62.92**	**63.40**	**63.70**	**53.18**	**62.62**
***Glycerolipids:***	Mono-acylglycerol (MAG)	22	0.3178	0.3356	0.3635	0.4066	0.3480
	Di-acylglycerol (DAG)	144	6.8538	7.3225	7.9311	8.0919	7.2712
	Tri-acylglycerol (TAG)	342	19.7011	18.8082	17.0849	25.0831	19.6508
	**Total:**	**508**	**26.87**	**26.47**	**25.38**	**33.58**	**27.27**
***Fatty acyls:***	(O-acyl) -1-hydroxy fatty acids (OAHFA)	32	7.5084	6.9170	8.3547	9.6297	7.6378
	**Total**	**32**	**7.51**	**6.92**	**8.35**	**9.63**	**7.64**
***Sphingolipids:***	Sphingomyelin (SM)	28	0.8976	1.0002	0.4264	0.3510	0.6737
	Sphingomyelin (phSM)	8	0.0113	0.0107	0.0101	0.0112	0.0089
	Sphingosine (So)	1	0.0032	0.0034	0.0117	0.0002	0.0014
	Ceramide (Cer)	58	1.0331	1.0468	1.0199	1.6783	0.8862
	Monosaccharide ceramides (CerG1)	1	0.0012	0.0012	0.0013	0.0549	0.0022
	Glycosylceramide series (CerG2GNAc1)	1	0.0708	0.0365	0.0657	0.0160	0.0288
	**Total**	**97**	**2.02**	**2.10**	**1.54**	**2.11**	**1.60**
***Saccharolipids:***	Di-galactosyl diacylglycerol (DG-DAG)	3	0.0147	0.2145	0.0118	0.0437	0.0217
	Mono-galactosyl diacylglycerol (MG-DAG)	31	0.5746	0.8062	0.8896	1.3348	0.7240
	Mono-galactosyl monoacylglycerol (MG-MAG)	4	0.0476	0.0515	0.0695	0.0322	0.0440
	Thio-isorhamnose diglyceride (SQDG)	2	0.0100	0.0079	0.0202	0.0416	0.0455
	**Total**	**40**	**0.65**	**1.08**	**0.99**	**1.45**	**0.84**
***Sterol lipids:***	Yeast sterol (ZyE)	2	0.0021	0.0023	0.0021	0.0037	0.0035
	Cholesterol esters (ChE)	5	0.0374	0.0391	0.0405	0.0415	0.0340
	**Total**	**7**	**0.04**	**0.04**	**0.04**	**0.05**	**0.04**
**Total lipid components**		**1286**	100

**Table 5:** Changes in the lipid-type distribution in produced tuna oils under different MW-SDME conditions.

**MW power (W)**	**Polar lipids (%)**				**Non-polar lipids (%)**	
	**PL**	**FFA**	**MAG**	**DAG**	**CHO**	**TAG**
**0(untreated)**	66.17 ± 1.23^a^	6.85 ± 0.16^c^	0.32 ± 0.04^a^	6.92 ± 0.22^d^	0.04 ± 0.00^a^	19.70 ± 0.21^b^
**200**	65.98 ± 0.89^a^	7.32 ± 0.13^bc^	0.34 ± 0.02^a^	7.51 ± 0.32^cd^	0.04 ± 0.01^a^	18.81 ± 0.18^c^
**400**	66.22 ± 1.07^a^	7.93 ± 0.17^ab^	0.36 ± 0.0^a^	8.35 ± 0.28^bc^	0.04 ± 0.01^a^	17.08 ± 0.12^c^
**600**	63.79 ± 0.85^a^	7.27 ± 0.25^bc^	0.35 ± 0.02^a^	8.94 ± 0.08^ab^	0.04 ± 0.00^a^	19.65 ± 0.32^b^
**800**	56.74 ± 0.43^b^	8.09 ± 0.22^a^	0.41 ± 0.03^a^	9.63 ± 0.20^a^	0.05 ± 0.00^a^	25.08 ± 1.21^a^

PL indicates phospholipids; FFA indicates free fatty acids; MAG indicates mono-acylglycerol; DAG indicates di-acylglycerol; TAG indicates tri-acylglycerol. CHO indicates cholesterol.

Noted different letter lower in superscript for results in each column illustrated significant differences among treatments (*P* < 0.05).

### 3.6 Principle components analysis evaluating correlations of tests and treatments

The correlation between all oil production efficiency and quality-related tests (oil recovery rate, POV, AV, *p*-AV, TOTOX, PL, TAG, DAG, MAG, EPA, and DHA) under different MW powers in MW-SDME treatments were analyzed by the principal component analysis (PCA) and presented in PCs correlations graph and correlation heatmap.

**Fig. 3:**
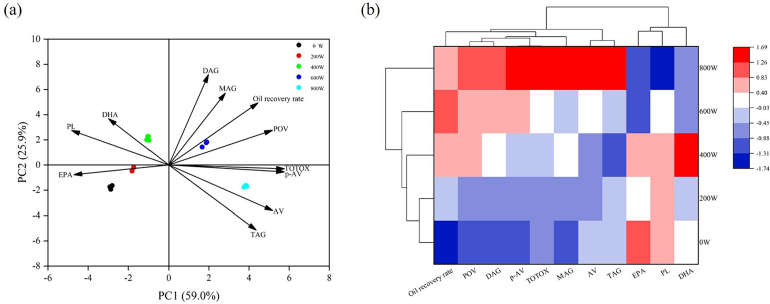
(a) Principal component analysis (PCA) of oil recovery rate, POV, TOTOX, PL, AV, *p*-AV, TAG, MAG, DAG, EPA, and DHA under different MW powers; (b) Correlation heatmap between above tests and MW powers under MW-SDME methods.

In Fig. 3a, the principal component 1 (PC1) and principal component 2 (PC2) in the analysis accounted for 59.0% and 25.9% of the total variance, respectively, total PCs in the analysis was up to 84.9%, indicating that it was a reliable PCA analysis. In addition to the low MW power group (200 W) and the untreated group (0 W) which were in the same quadrant, all other groups were found located in the other three different quadrants. This indicates that different MW power intensities affect the general extraction efficiency and qualities of fish oil under MW-SDME methods. Besides, fish oil produced from 400 W-SDME had a strong correlation with higher retention of DHA and PL content in produced oils. The fish oil produced by the medium-to-high MW power group (600 W) was found to be better correlated with the oil recovery rate, hydrolyzed triglycerides proportion (DAG and MAG), and the initial oxidation products of oil (POV). There was a strong correlation between high microwave power group fish oil (800 W-SDME) and lipid oxidation index (AV, *p*-AV, TOTOX), which was directly attributed to the high TAG content in this group, which was more prone to lipid deterioration.

Fig. 3b is the correlation heatmap under further analysis. It shows the correlation between MW powers (0, 200, 400, 600, and 800 W) and all tests. Obviously, fish oil produced by method without MW (SDME only) and 200 W-SDME was positively correlated with higher EPA content but more emulsifying PL content, negatively correlated with oil recovery rate and lipid oxidation indexes such as POV and AV. 400 W-SDME was found to have a higher expression with DHA content and negatively correlated with TAG and AV value. This condition is suggested to produce high DHA fish oil for a particular purpose. The fish oil produced from the medium-to-high MW power group (600 W) had the opposite trend with the lower power group in consideration of lipid composition, it has a higher expression on oil recovery rate and the initial oxidation products of oil (POV). Compared with other groups, the fish oil in the high microwave power group (800 W-SDME) had a large proportion of glycerides and a large correlation with the oil oxidation index. It is not recommended to use 800 W-SDME in the production of high-quality tuna liver oil. In general, 600 W-SDME may be a recommended condition with high oil recovery efficiency and good stability, and medium and low microwave power pretreatment may be the best choice to obtain high DHA fish oil.

### 3.7 Microstructural disruption observation

**Fig. 4:**
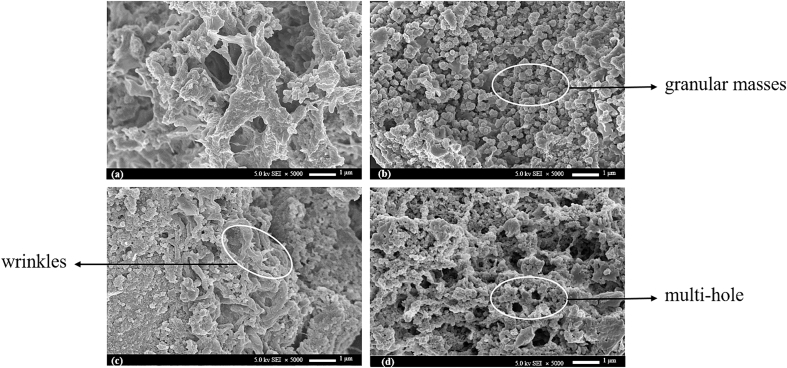
Scanning electron microscope (SEM) images on tuna liver tissues under different studied treatment groups including (a)freshly collected liver materials from the industry processing line; (b)600 W microwave pretreated liver materials (MW); (c)SDME extracted liver materials, and (d)MW-SDME extracted liver materials with ×5000 magnification.

Based on the SEM images of all four different groups in Fig. 4.Considering higher MV can be rapidly converted into heat energy, rapidly raising the intracellular temperature, thus resulting in effective tissue damage (Hu et al., 2019). In specific, in comparison with the control group (Fig. 4a), After 600 W MW pretreatment, many granular, irregular small masses were created on the surface (Fig. 4b), which might be caused by the thermal degradation of tuna liver matrix proteins(Costa & Bragagnolo, 2016). Furthermore, compared with untreated fish liver (Fig. 4a), the application of SDME (Fig. 4c) induced a significant dehydration effect and produced many folds on the surface of the liver material. This finding proved that DME having both hydrophilic and lipophilic properties under subcritical extraction, can extract all juices (water and oil) from the material at the same time. By combining SDME and MW pretreatment during the liver oil production (Fig. 4d), the left-over liver materials tissues got loose and became more porous, and the extended degree of protein denaturation was formed, indicating MW-SDME was a promising technology for releasing fish oils from tuna livers with effective disruption on tissue protein structure. It was consistent with our oil recovery and quality results.

## 4 Conclusions

This study investigated the effect of different MW power on quality characteristics and changes in lipids of fish oil under subcritical extraction. The recovery rate of fish oil increased first with the increase of microwave power and then decreased due to coking caused by high power, and reached the maximum (93.12%) at 600 W. The process parameters for the maximum recovery rate of fish oil were 600 W, extraction for 100 min, and a solid-liquid ratio of 1: 4 g/mL. The POV, AV, *p*-AV, and TOTOX of fish oil are increased with an increase in MW power. Significant differences from MW powers were proved in oil stability, ω-3 fatty acids composition, and lipid types regarding polarity. 400 W-SDME might be suitable for high DHA oil production. LC-MS / MS analysis showed that with the increase of microwave power, the main lipid components DAG, TAG, PL, and MAG of tuna oil changed. At 400 W, the PL and DHA contents of fish oil were higher. At 600 W, the relative contents of DAG and MAG were higher. At 800 W, the content of TAG reached the highest, indicating that lipid oxidation occurred in fish oil. Correlation analysis also showed that fish oil in the 800 W treatment group had a greater correlation with oxidation indexes such as AV, *p*-AV, and TOTOX. Microstructure analysis showed that microwaves combined with subcritical technology could effectively destroy the structure of matrix protein in tuna livers, to better promote the release of oil. The MW-SDME on tuna livers represents an innovative technology for obtaining high-value-added products of tuna oil from canned tuna. This study shows that microwave pretreatment combined with subcritical extraction can obtain high-quality and high recovery rate oil.

## CRediT authorship contribution statement

**Wenjie Wang:** Writing – review & editing, Supervision, Funding acquisition. **Yuliang Xiao:** Writing – original draft, Investigation, Formal analysis. **Yicheng Ding:** Writing – review & editing, Data curation. **Yihong Li:** Methodology, Investigation, Formal analysis. **Yihua Zhu:** Writing – review & editing. **Xuxia Zhou:** Writing – review & editing, Supervision, Funding acquisition.

## Declaration of competing interest

The authors declare that they have no known competing financial interests or personal relationships that could have appeared to influence the work reported in this paper.

## Data Availability

No data was used for the research described in the article.
